# A case of surgical pulmonary artery plasty for pulmonary artery stenosis after right single-lung transplantation

**DOI:** 10.1186/s44215-022-00026-x

**Published:** 2023-04-05

**Authors:** Hideki Nagata, Takashi Kanou, Eriko Fukui, Toru Kimura, Naoko Ose, Soichiro Funaki, Masato Minami, Masaki Taira, Takayoshi Ueno, Shigeru Miyagawa, Yasushi Shintani

**Affiliations:** 1 Department of General Thoracic Surgery, Graduate School of Medicine, Osaka University, Suita, Osaka Japan; 2 Department of Cardiovascular Surgery, Graduate School of Medicine, Osaka University, Suita, Osaka Japan

**Keywords:** Lung transplantation, Pulmonary artery stenosis, Pulmonary artery plasty

## Abstract

**Background:**

In lung transplantation, vascular complications are relatively rare. However, severe pulmonary artery stenosis after lung transplantation can cause clinical symptoms and lead to graft dysfunction.

**Case presentation:**

The present patient underwent right lung transplantation for severe chronic obstructive pulmonary disease. After lung transplantation, pulmonary blood flow scintigraphy showed a decreased blood flow to the transplanted lung, and contrast-enhanced computed tomography showed pulmonary artery anastomotic stenosis. As the initial treatment, balloon dilation of the pulmonary artery was attempted and unsuccessful. Right pulmonary artery plasty was then performed 5 months after lung transplantation. Following the procedure, the pulmonary artery stenosis was released, and the blood flow to the transplanted lung improved.

**Conclusion:**

Surgical treatment should be considered for pulmonary artery stenosis after lung transplantation if minimally invasive intervention is not effective.

## Background

In lung transplantation, pulmonary artery stenosis at the anastomosis site is rare. The incidence rate is less than 1% [1], and most cases do not show clinical symptoms. However, in severe cases, therapeutic intervention is required to improve the clinical course [1].

We herein report a 60-year-old man with pulmonary artery stenosis after right single-lung transplantation who underwent successful pulmonary artery plasty with dramatic improvement in his condition.

## Case presentation

Cadaveric right lung transplantation was performed for a 60-year-old man with severe chronic obstructive pulmonary disease. Based on the calculation from the predicted vital capacity of the donor and recipient, the graft lung was 13% undersized for the recipient. However, the status of recipient was deteriorated rapidly, so we were urged to perform lung transplantation. Veno-arterial extracorporeal membrane oxygenation (VA-ECMO) was established through the left femoral artery and vein. The patient was set in the lateral position, and an anterolateral incision was selected. The bronchial anastomosis was performed in a normal position and the donor left atrial cuff was anastomosed with the recipient’s left atrial cuff. Then the pulmonary artery (PA) was anastomosed by continuous closure with 5–0 monofilament polypropylene sutures.

**Fig. 1:**
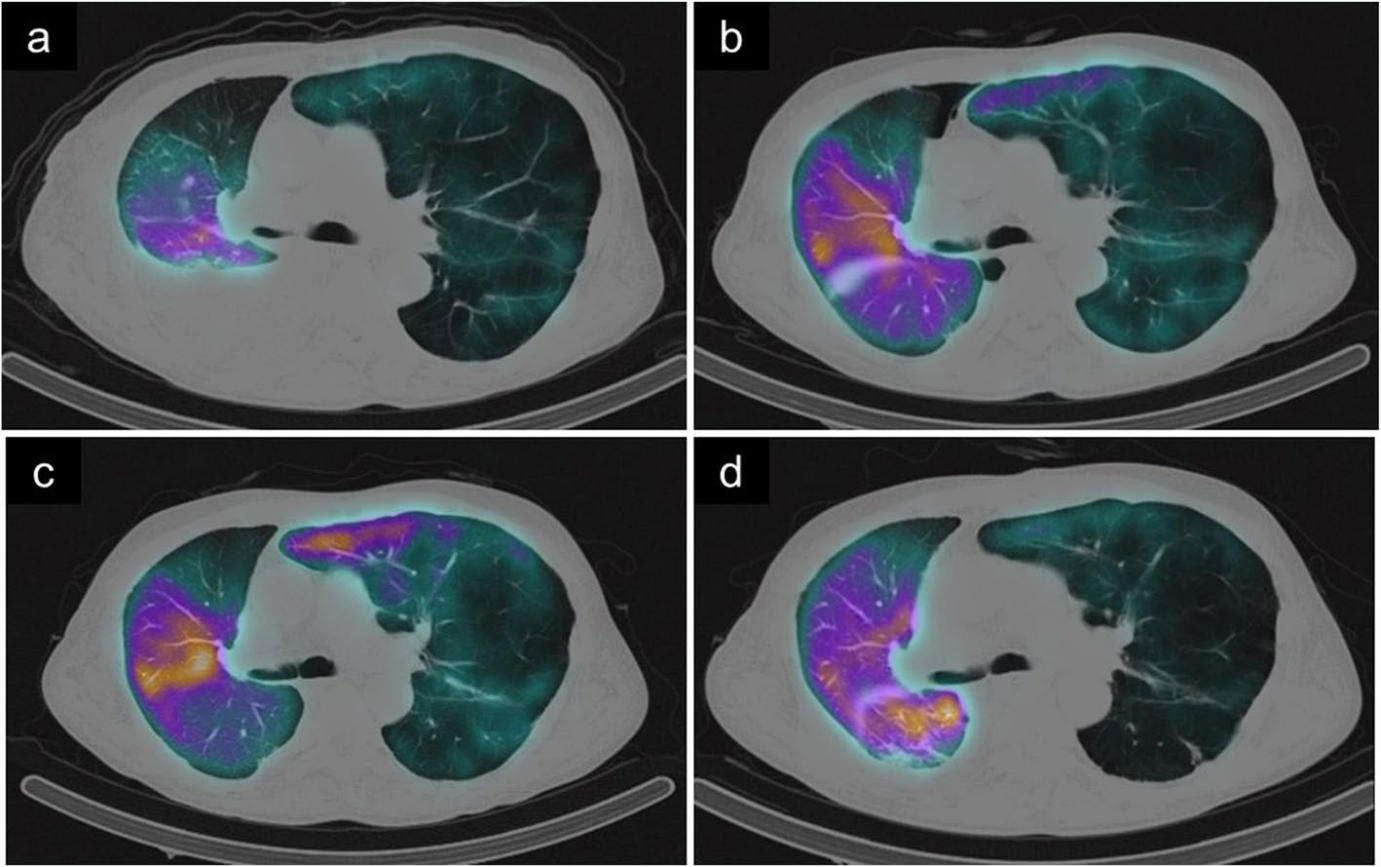
Pulmonary perfusion scintigraphy after right lung transplantation and pulmonary artery plasty. The pulmonary right-to-left blood flow ratio by pulmonary perfusion scintigraphy was 37:63 at 2 weeks after transplantation (**a**) and 49:51 at 7 weeks after transplantation (**b**). However, at 10 weeks, the flow to the transplanted lung decreased, showing a ratio of 43:57 (**c**). At 5 weeks after pulmonary artery plasty, pulmonary perfusion scintigraphy showed an increased flow to the right lung, with a right-to-left ratio of 67:33 (**d**)

**Fig. 2:**
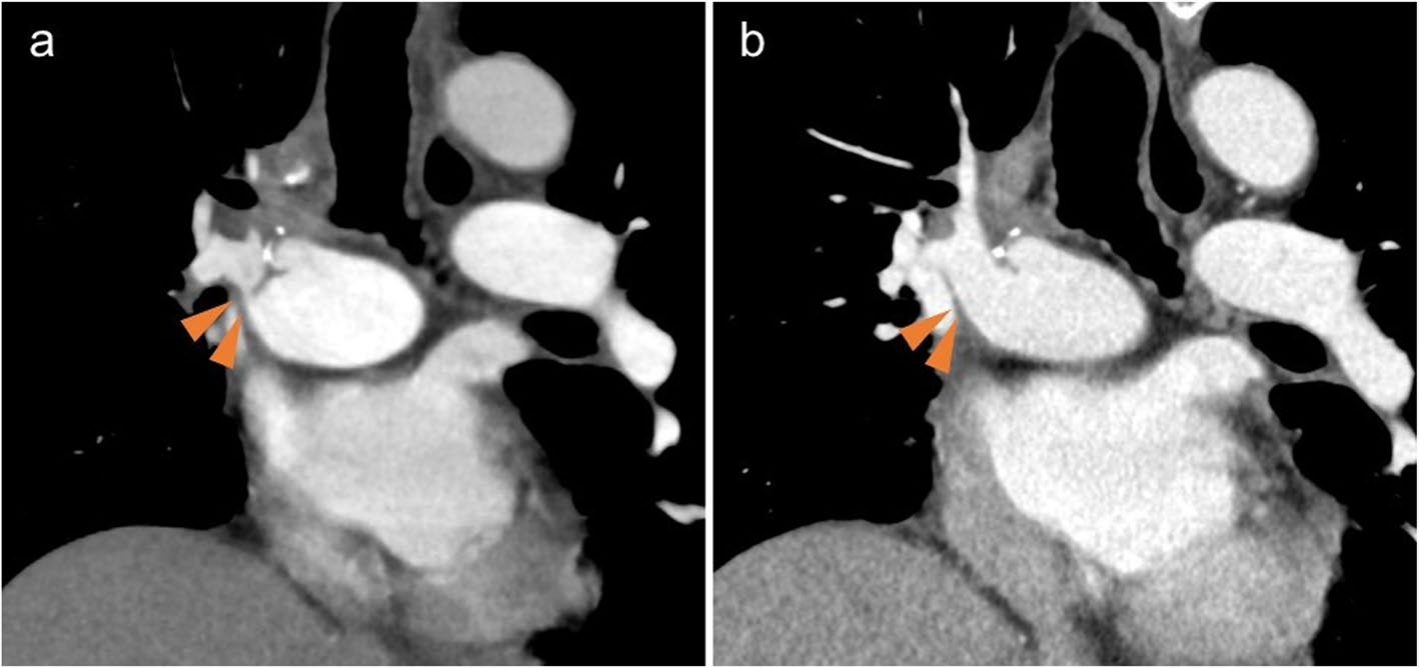
Contrast-enhanced CT after right lung transplantation and pulmonary artery plasty. Contrast-enhanced CT showed prominent stenosis at the anastomosis of the right pulmonary artery. Arrows indicate stenosis site (**a**). Contrast-enhanced CT after pulmonary artery plasty showed relief of the stenosis of the right pulmonary artery (**b**)

Due to the large caliber difference in the PA between the donor and recipient, the recipient’s right interlobal PA was anastomosed with the main PA of the donor lung. The right-to-left pulmonary blood flow ratio on pulmonary perfusion scintigraphy was 37:63 at 2 weeks after transplantation and 49:51 at 7 weeks, showing an increased blood flow to the transplanted right lung (Fig. 1a, b). However, dyspnea on exertion appeared from 10 weeks after transplantation, and the SpO_2_, which had previously been ≥ 95% on 0.5 L of oxygen, dropped below 95%, even on 1.5 L of oxygen. Pulmonary perfusion scintigraphy showed a decreased blood flow in the right lung (right:left = 43:57) (Fig. 1c). Contrast-enhanced computed tomography (CT) showed prominent stenosis at the anastomosis of the right PA (Fig. 2a).

**Fig. 3:**
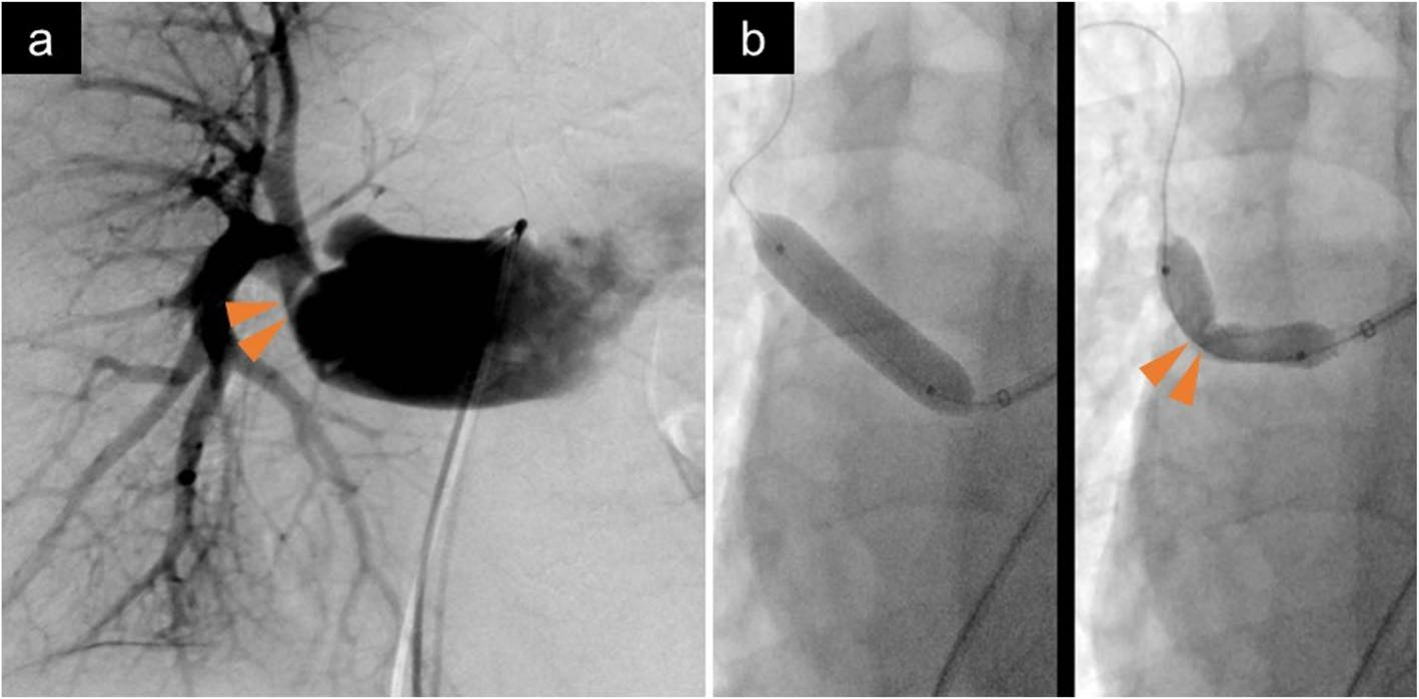
Pulmonary artery angiography by a right heart catheter examination. The right heart catheter showed stenosis at the anastomosis of the right pulmonary artery trunk. Arrows indicate stenosis site (**a**). The stenosis was not relieved by balloon dilation (**b**)

To resolve the stenosis, pulmonary angiography and balloon dilation under right heart catheterization was performed at 15 weeks after transplantation. The right heart catheter indicated a differential in the right PA pressure; the pressure proximal to the stenosis in the right PA was 40/16 (mean 27) mmHg, while that distal to the stenosis was 18/11 (mean 14) mmHg. Although balloon dilation was attempted, the stenosis was not relieved (Fig. 3). Based on these results, surgical right PA plasty was performed 5 months after lung transplantation.

**Fig. 4:**
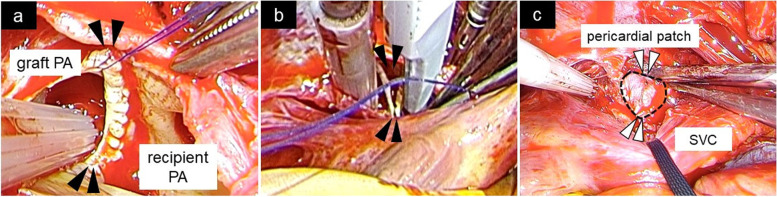
Intraoperative findings of right pulmonary artery. The pulmonary artery (PA) wall was incised at the anastomosis and the upper portion of PA wall was thickened and protruded into the lumen. Arrows indicate thickened pulmonary artery walls (**a**). This thickened PA wall was resected (**b**) and the resected wall section was anastomosed between the graft and the recipient's PA wall. The initially incised PA wall was reconstructed with an autologous pericardial patch. Arrows and dotted lines indicate the pericardial patch (**c**)

We chose the supine, midline sternal incision approach. After establishing cardiopulmonary bypass, PA plasty was performed without both cardiac arrest and PA clamp. The anastomosis of the right PA was identified behind the superior vena cava (SVC), which was tractioned to the left, while the wall of the right PA was incised at the proximal portion of the anastomosis. At the anastomosis, the upper portion of the PA wall was thickened and protruded into the lumen. This thickened area was just at the pulmonary artery anastomosis (Fig. 4). The incision of the PA wall was extended to the distal side of the anastomosis, and the thickened PA wall was excised across the anastomosis. The excised thickened wall section was anastomosed between the graft and the recipient’s PA wall. Autologous pericardium was applied as the patch closure of initial PA incision.

Following the procedure, the patient was weaned from the artificial heart–lung machine and returned to the intensive-care unit. He was weaned from the ventilator on postoperative day 9. Pulmonary blood flow scintigraphy at 5 weeks postoperatively showed an improvement in the blood flow to the transplanted lung, with a right-to-left ratio of 67:33 (Fig. 1d). Contrast-enhanced CT showed that the PA stenosis was resolved (Fig. 2b). The dyspnea on exertion and decreased SpO_2_ also improved. The patient was transferred to a rehabilitation center 9 weeks after the second surgery.

## Discussion

Vascular-related complication after lung transplantation are rare, occurring in 0.3–1.0% of patients [1–3]. In most cases, PA stenosis after lung transplantation is not severe, and symptoms do not appear [1]. However, when the diameter of the anastomosis is narrowed to less than 75% of the anastomotic vessel, therapeutic intervention is required due to the appearance of symptoms and pulmonary insufficiency [1, 4, 5]. In past reports, PA anastomotic stenosis was reported to occur at various times, from four days to 6 years after transplantation [1, 3, 6–9], so attention is needed in not only the early stage but also the late stage after transplantation. Contrast-enhanced CT and pulmonary arteriography are useful for making a diagnosis [1, 6], and indeed, in the present case, PA anastomotic stenosis was diagnosed by contrast-enhanced CT at 12 weeks after transplantation.

Generally, the treatment strategy for PA stenosis after lung transplantation includes surgical repair and percutaneous angioplasty. In recent years, there have been an increasing number of reports on the success of percutaneous angioplasty. Two successful case reports showed the efficacy of stenting and balloon dilatation for PA stenosis; the technical success rate was 100% in both cases, and complications were observed in 1 of 5 cases and 0 of 12 cases [1, 6]. Those percutaneous interventions are considered minimally invasive procedures. In contrast, surgical PA angioplasty is a highly invasive procedure that carries a high risk of complications under the usage of immunosuppressive drugs. There are few reports of success cases of open repair of PA stenosis after lung transplantation. In our case, percutaneous arteriography was performed first, and then balloon dilation was attempted for the stenotic area, which was unsuccessful; surgical PA angioplasty was thus eventually required.

One cause of PA stenosis in the present case was thought to be size-mismatch between donor and recipient. On reviewing the imaging findings, dyspnea on exertion appeared around the time the air space at the right lung apex disappeared on chest X-ray. Our hypothesis is that once the donor lung fully expanded, it caused a shift in the hilum structure, resulting in bending and stenosis of the PA. Banerje et al. reported a similar case of twisting of the anastomosis with expansion of the transplanted lung [10]. In our case, because the PA diameter of the donor lung was smaller than expected, the anastomosis was distal to the recipient’s trunk superior bifurcation in order to match the recipient's PA diameter. This might have resulted in a situation where the distance from the pulmonary hilum to the PA anastomosis was longer than usual. We suspect that a longer distance from the pulmonary hilum to the anastomosis of the PA allows bending to occur more easily. To prevent such complications, the vessel diameter should be corrected, such as with a tack suture [11], and the PAs should be anastomosed at the main pulmonary trunk.

## Conclusion

We experienced a case of PA stenosis caused by bending at the PA anastomosis after right single-lung transplantation. The PA should be anastomosed at the main pulmonary trunk as much as possible even if there is a caliber difference between donor PA and recipient PA, and surgical repair is still considered effective against severe stenosis at the PA anastomosis.

## Data Availability

The datasets during and/or analyzed during the current study available from the corresponding author on reasonable request.

## Abbreviations

VA-ECMO: Veno-arterial extracorporeal membrane oxygenation
PA: Pulmonary artery
CT: Computed tomography
SVC: Superior vena cava
